# Effects of Physical Exercise Interventions on Spatial Orientation in Children and Adolescents: A Systematic Scoping Review

**DOI:** 10.3389/fspor.2021.664640

**Published:** 2021-06-17

**Authors:** Christina Morawietz, Thomas Muehlbauer

**Affiliations:** Division of Movement and Training Sciences/Biomechanics of Sport, University of Duisburg-Essen, Essen, Germany

**Keywords:** cognitive functioning, orientation, navigation, youth, physical exercise

## Abstract

**Background:** Regular physical exercise plays an integral part in the psychomotor and psychosocial development of children and adolescents, with complex motor and cognitive processes closely linked. Spatial abilities, one aspect of cognitive functioning start to evolve from earliest childhood and reach adult-like levels by early adolescence. As they have been associated with good spatial orientation, wayfinding, map-reading skills, problem solving or analyzing spatial information, these skills facilitate independence and autonomy while growing up. Despite promising results, only few studies investigate this relation between physical exercise and spatial abilities. To use this benefit and develop purposive physical exercise interventions, it is essential to summarize the current evidence.

**Objectives:** This literature review aims to systematically summarize findings regarding the impact of physical exercise interventions on spatial abilities in healthy children and adolescents and identify knowledge gaps.

**Methods:** A systematic search of the literature according to the PRISMA guidelines was conducted on the databases Pubmed, Web of Science, Cochrane Library, SportDiscus, and PsycInfo from their inception date till March 2021. Additionally, Google Scholar and refence lists of relevant publications were searched. A descriptive analysis of results was conducted.

**Results:** The literature search identified a total of *N* = 1,215 records, 11 of which met the inclusion criteria and were analyzed in this review. A total of 621 participants aged 4 to 15 years participated in the studies. Exercise interventions included sport-specific activities, motor-coordinative exercises, high-intensity functional training or spatial orientation/navigation training. Five studies evaluated training effects on mental rotation performance (i.e., Mental Rotation Test), four studies investigated visuo-spatial working memory function/spatial memory (i.e., Corsi Block Test, Virtual Reality Morris Water Maze) and two studies tested spatial orientation capacity (i.e., Orientation-Running Test). Overall, results show a potential for improvement of spatial abilities through physical exercise interventions. However, keeping the diversity of study designs, populations and outcomes in mind, findings need to be interpreted with care.

**Conclusions:** Despite growing interest on the effects of physical exercise interventions on spatial abilities and promising findings of available studies, evidence to date remains limited. Future research is needed to establish how spatial ability development of healthy children and adolescents can be positively supported.

## Introduction

In today's increasingly sedentary society, rising levels of physical inactivity have been associated with spiking health care expenses due to a growing prevalence in non-communicable diseases like cardiovascular disease, diabetes, overweight/obesity, musculoskeletal- or mental health problems (World Health Organization, 2010). Regular physical activity has not only shown to reduce these risk factors and to be beneficial for general health behavior but appears to considerably facilitate psychomotor and psychosocial development from childhood on (Shephard, 1997; World Health Organization, 2010). Results of a study by Frick et al. (2009) claim that particularly in children motor processes play an integral part in the development of cognitive functions. Recent systematic reviews disclose emerging evidence on the positive association between increased physical activity or physical fitness and aspects of academic achievement as well as cognition (Singh et al., 2019; Alvarez-Bueno et al., 2020). In particular performance in mathematics but also skills related to language or reading appear to benefit from higher levels of physical fitness in children (Chaddock-Heyman et al., 2015; Alvarez-Bueno et al., 2020). Even though more research is needed to specify the impact of physical activity in general and physical exercise in particular on overall academic performance (van der Fels et al., 2015; Donnelly et al., 2016; Singh et al., 2019), there seems to be agreement on the relationship of complex cognitive and motor skills at prepubescent age (van der Fels et al., 2015) as well as the implication of physical activity and physical exercise on the development of these functions at preschool age (Zeng et al., 2017). It is therefore of highest importance to promote these motor activities from early age on in order to facilitate adequate motor and cognitive development throughout childhood and adolescence (Timmons et al., 2007; World Health Organization, 2010). Throughout this review, the terms “physical activity” and “physical exercise” will be used as specified in the following definition by Caspersen et al. (1985): “Physical activity is defined as any bodily movement produced by skeletal muscles that results in energy expenditure. The energy expenditure can be measured in kilocalories. Physical activity in daily life can be categorized into occupational, sports, conditioning, household, or other activities. Exercise is a subset of physical activity that is planned, structured, and repetitive and has as a final or an intermediate objective the improvement or maintenance of physical fitness. Physical fitness is a set of attributes that are either health- or skill-related. The degree to which people have these attributes can be measured with specific tests.”

Spatial abilities, as one aspect of complex cognitive functioning, start to evolve from earliest childhood on (Fernandez-Baizan et al., 2019). Throughout lifetime, these demanding skills go through various phases of development (Newcombe, 2019) with age-appropriate spatial abilities being indicative of normal brain development (Leplow et al., 2003). Due to growing motor abilities and increasing motor independence, infants start to explore, interpret and interact with their surroundings, which aids the formation of spatial perception and knowledge (Farran et al., 2019). Already from the age of four, the capability to mentally rotate stimuli becomes apparent (Jansen and Heil, 2010; Frick et al., 2013a). Spatial abilities continue to develop throughout childhood (Farran et al., 2019; Newcombe, 2019) and reach an adult-like level by early adolescence (Newcombe, 2019). While remaining stable in adolescents and adults, some aspects of spatial performance, like the formation and use of cognitive maps, have been found to deteriorate again in the elderly population (Iaria et al., 2008; Head and Isom, 2010). Spatial abilities have been associated with numerous aspects of humans' everyday life. Amongst others, good spatial abilities have been linked to high orientation and navigation capabilities, map reading skills, remembering object locations, perceiving spatial relations of objects in relation to one another and in space, problem solving or analyzing and interpreting spatial information (Tzuriel and Egozi, 2010; Fernandez-Baizan et al., 2019). Particularly orientating oneself and finding one's way through the known and unknown environment are essential skills to gain independence and lead an autonomous life (Claessen et al., 2016; Fernandez-Baizan et al., 2019).

With good spatial abilities seemingly providing a foundation for a successful and independent life, this development should be encouraged consistently from infancy onwards. Therefore, it is surprising that spatial abilities are not routinely taught at school yet (Wai et al., 2009; Lowrie et al., 2018). There have been various successful attempts to improve spatial competencies in children with theoretical paper-and-pencil-, computer- or classroom-based approaches (Hawes et al., 2015; Lowrie et al., 2017). However, only few studies make use of the link between cognitive and motor competences and attempt to evaluate the relation between physical exercise and spatial abilities. Here, mental rotation (MR) is the most frequently researched spatial ability (Hoyek et al., 2014; Heppe et al., 2016). Hoyek et al. (2014) point out the positive correlation between children's MR performance and the time they need to complete an obstacle chase. They did, however, not find an association between MR performance and sprinting time which indicates that the type of physical exercise might play an important role in the development of spatial abilities. A study by Jansen and Heil (2010) revealed that accomplishment in a MR task can be predicted by the level of motor control and intelligence in 5 and 6 year old children. Other research found that students whose curriculum included larger amounts of physical education (PE) classes performed significantly better on a MR task than students with normal levels of PE (Jansen et al., 2018). In line with these findings, another study established larger short-term improvements in MR following 1 h of creative dance training compared to regular PE (Jansen and Richter, 2015). Reasons for the beneficial effect of increased physical activity levels or higher physical fitness on spatial abilities in children and adolescents are believed to lie in neurophysiological adaptation mechanisms that occur in the developing brain, particularly in areas of the brain closely related to spatial abilities. Amongst others, increased cerebral blood flow that could lead to improved provision of oxygen and nutrients (Chaddock-Heyman et al., 2016), a higher production of growth factors facilitating angiogenesis and neurogenesis (Jeon and Ha, 2017), upregulation of neurotransmitters and a variety of alterations in brain structure and function (Chaddock-Heyman et al., 2013, 2018, 2020; Esteban-Cornejo et al., 2019) have been associated with higher physical activity. The subsequent cerebral adaptations are expected to facilitate the development and functioning of brain and cognition like activation patterns, information processing, attention, cognitive flexibility and control, working memory or visuo-spatial functioning (Singh et al., 2019; Valkenborghs et al., 2019; Meijer et al., 2020) which can be related to spatial abilities.

Despite these encouraging findings and the importance of physical exercise and spatial abilities for the various aspects of a well-balanced life, to date only little is known about the impact of motor training long-term interventions on spatial orientation and spatial abilities in youth. While these studies reveal a correlation between physical exercise and spatial abilities in children and adolescents, no causal relationship in line of cause (i.e., implementation of a physical exercise intervention) and effect (i.e., adjustment of spatial abilities) can be derived from these cross-sectional findings. Merely the evaluation of intervention studies consisting of experimental and control conditions allows for a statement regarding the effect of physical exercise interventions on spatial abilities. Identification of the most suitable physical exercise approaches could provide the opportunity to not only improve physical fitness and motor skills in children and adolescents but at the same time enhance spatial abilities and orientation. Thus, aiming to identify research gaps and to facilitate future research in this field, this scoping review systematically reports the current body of evidence on physical exercise interventions to enhance spatial orientation in a young and healthy population.

## Methods

A systematic scoping review of the literature was conducted to in accordance with the PRISMA Extension for Scoping Reviews (PRISMA-ScR) (Tricco et al., 2018). In line with Munn et al. (2018), this approach was selected aiming to provide an overview of the extend of current evidence in this emerging, little studied field of research and to identify and analyze current gaps in knowledge. Like systematic reviews, scoping reviews are conducted in a structured and reproducible manner with the purpose to diminish bias (Munn et al., 2018). In contrast to systematic reviews, a scoping review might cover a relatively broad research question and rarely includes a critical appraisal or risk of bias assessment of the included research in order to outline the current scope of literature (Arksey and O'Malley, 2005; Peters et al., 2015). Therefore, no risk of bias assessment and critical appraisal of the literature was conducted in this present systematic scoping review.

### Search Strategy

**Figure 1:**
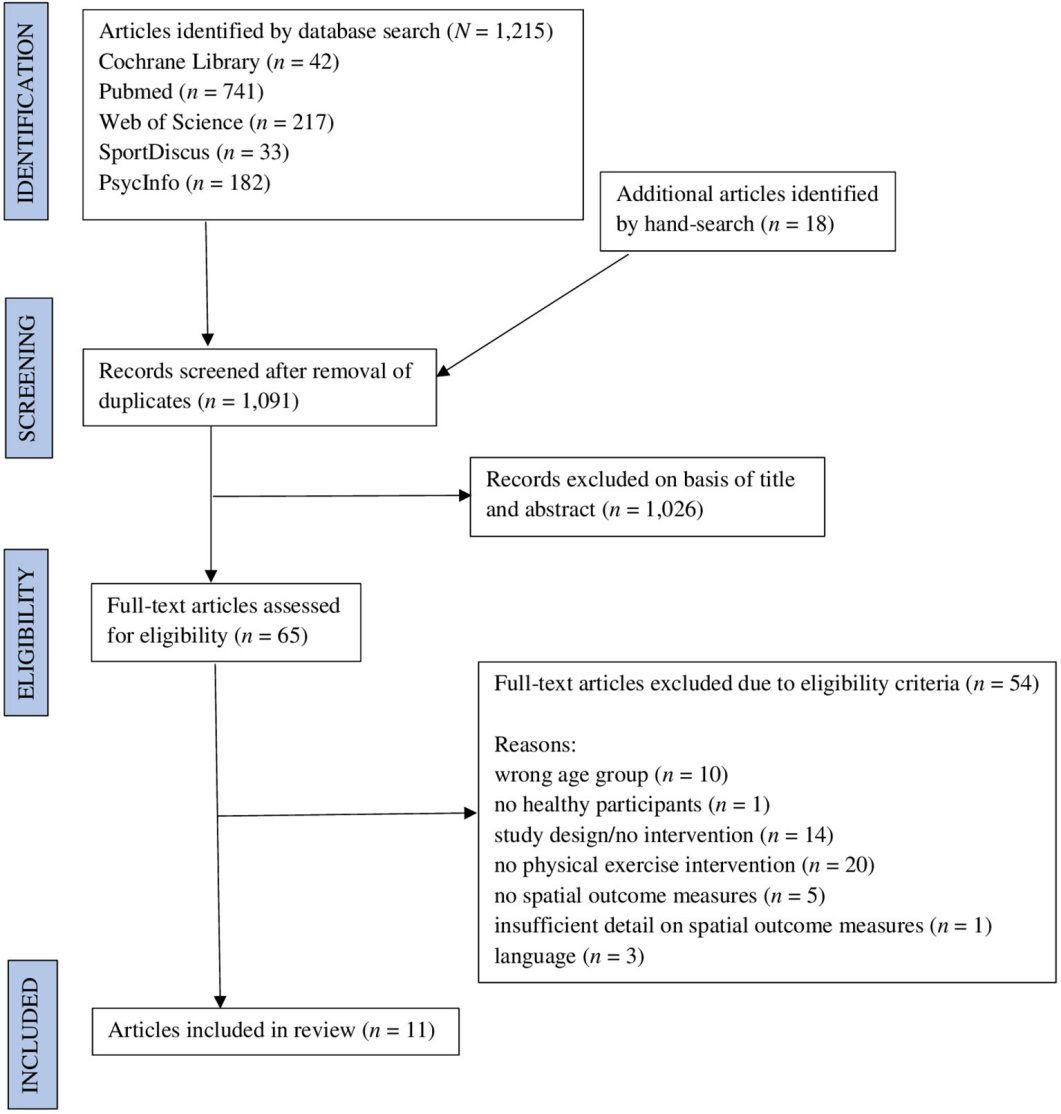
PRISMA flow diagram.

The electronic databases Pubmed, Web of Science, Cochrane Library, SportDiscus, and PsycInfo were searched systematically to identify relevant literature. A coherent search strategy was developed and refined in accordance with the PICOS-scheme. All search results were exported into EndNote (version X9 Bld 12062) and duplicates were removed. The remaining citations were then uploaded to Rayyan QCRI (Ouzzani et al., 2016), a free of charge web application aiding preparation of systematic reviews. Titles and abstracts of all records were screened independently by both authors for eligibility according to the inclusion and exclusion criteria. Full-text versions of articles were retrieved and screened for eligibility if the information provided in the title and abstract was insufficient. Subsequently, full-text versions of all potentially relevant studies were obtained and assessed for inclusion by both reviewers. Disagreements were resolved by discussion and consensus. The study selection process and reasons for exclusion of records are documented in the PRISMA 2009 Flow Diagram (Moher et al., 2009; Figure 1). An example of the final search string for the inquiry in the Web of Science database is depicted in a (Supplementary Material 1). Systematic searches were conducted from inception of the respective database up until March 2021. No search filters were applied to any of the searches. Reference lists of identified records and review articles in the associated research field were hand-searched to detect additional relevant publications that were not identified by the databases. Additionally, Google Scholar was searched for additional literature.

### Data Charting

The data charting process was documented with a modified version of the “Data collection form for intervention reviews: RCTs and non-RCTs” (The Cochrane Collaboration, 2014) by both authors. In an iterative process, the form was refined, and data were updated continuously to capture all relevant information.

### Inclusion and Exclusion Criteria

Studies were eligible for this review if they met the predefined inclusion criteria and provided sufficient information with respect to the PICOS (population, intervention, comparison, outcomes, study design) search approach (Tricco et al., 2018). Criteria for inclusion were as follows: (i) population: healthy children (4–12 years) and/or adolescents (13–18 years); (ii) intervention: a physical exercise intervention of at least three sessions; (iii) comparison: include an active or a passive control group (e.g., age-matched participants that continued their regular physical exercise regimen, performed a different training or no training); (iv) outcomes: report at least one spatial orientation or MR or visuo-spatial working memory/spatial memory outcome; (v) study design: controlled studies with pre- and post-measurements; (vi) published in English or German language/literature; (vii) be available in full-text version. Studies were excluded if they: (i) involved only participants with pathological/developmental impairments; (ii) employed interventions without physical exercise (i.e., solely paper-and-pencil or computer-based training); (iii) evaluated training effects without control group; (iv) did not implement any measure of spatial orientation or MR or visuo-spatial working memory/spatial memory; (v) were books, reviews, editorials, uncontrolled studies, single case studies, cross-sectional studies, conference abstracts, or interviews.

### Data Extraction

Data were extracted on eligible author(s), year of publication, type of the study, study aim, participants involved, intervention type and parameters, outcome measures, drop-out rates, main findings, and self-reported limitations. Data were listed as mean and standard deviation (SD) if reported. Data retrieved by both authors were compared, and discrepancies were resolved by discussion and consensus.

### Synthesis of Results

Studies were grouped according to their main spatial outcome measure, i.e., Mental Rotation Test (mental rotation), Corsi Block Test and Virtual Reality Morris Water Maze (visuo-spatial working memory/spatial memory), and Running-Orientation Test (spatial orientation). For each group, population characteristics, interventions, and their modalities as well as outcome measures were summarized.

## Results

### Selection of Studies

Figure 1 provides an overview of the study selection process. The systematic search of the databases PubMed, Web of Science, Cochrane Library, SportDiscus, PsycInfo, and Google Scholar resulted in 1,215 articles for appraisal. Another 18 records were identified by hand-search. After removal of duplicates and screening of titles and abstracts, 65 articles were considered for further examination. Scrutinizing the full-text versions of these articles for eligibility, another 54 records were excluded for the following reasons: participants in ten studies were older than 18 years, one article did not involve any healthy participants, 14 records did not directly conduct an intervention, 20 records did not implement a physical exercise intervention approach, five studies did not meet the criteria for outcome measures, one article did not provide sufficient information on the spatial outcomes used and this information could not be retrieved from the author and three records did not meet the language requirements. The remaining eleven studies met all of our inclusion criteria and were analyzed in this systematic scoping review.

### Study Characteristics

Overall characteristics of the included studies are presented in Table 1. The table provides information on author(s), publication year, participant characteristics, interventions performed, and their modalities as well as outcome measures, measurement parameters and results.

**Table 1:** Overview of included studies.

**References**	**Participant characteristics**				**Groups; Intervention type**	**Training modalities**			**Outcome measures (parameters)**	**Results**
	***N* (IG/CG)**	**Sex (m/f)**	**Age, yrs (mean)**	**Training status (type of sport)**		**TP**	**TF**	**TD**		
**Mental rotation (mental rotation test)**										
Jansen et al. (2011a)	50 (26/24)	0/50	6–14 (IG: 10.4) (CG: 10.5)	Trained (gymnastics)	IG: juggling training (Rehoruli's method) + individual training at home CG: light strength training with elastic resistance bands (biceps curls, knee bend) (individual training at home)	13	IG: 2 + 7 CG: 7	IG: 30 + 10 CG: 10	(Computer) Mental Rotation Test (MRT) with 3-D block figures (18 line-drawings composed of 10 cubes; same stimuli for pre- and post-test): 432 trials in ~50 min. (+54 unrecorded test trials); reaction time	No sig. pre-post-improvements in MR error rate in IG and CG; no sig. between group differences sig. correlation of training effects and age in IG and CG; no sig. correlation with difference scores in RT or with error rates in IG and CG Analysis of correct MRT responses only: sig. faster RT at 90 and 180° disparity in IG compared to CG; sig. highest mean RT improvement at 90° for IG Sig. impact of age, not motor performance on MRT in IG
									IG: no. of successful throws CG: no. of knee bends	Sig. pre-post-improvement in motor performance in IG and CG
Bluechel et al. (2013)	84 (42/42)	36/48	8–10 (9.06)	Untrained	IG: specific coordinative motor training (bouncing task, handball task, motor memory game, orientating task, skateboard task, juggling task, catching ball task, catching task) CG: normal classroom lessons (no additional training)	2	IG: 5 CG: n/a	IG: 20 CG: n/a	(Paper-Pencil) MRT with 3-D block figures [Version A (Peters et al., 1995); redrawn version of the Vandenberg and Kuse MRT (Vandenberg and Kuse, 1978)]: 2 sets of 12 tasks; time-limit: 3 min. per set, 20 s break Questionnaire (latest grades in the subjects of Mathematics, German, Sports, Music, and Science)	Sig. main effect of time on MRT with larger improvements in IG; sig. time × group interaction; sig. between group difference at post-test in favor of IG; sig. main effect of gender with better MRT performance of boys than girls Correlation of mathematics grades and MR performance; no between group or gender differences in mathematics grades; no group × gender interaction
Jansen et al. (2013)	65 (44/21)	34/31	7–9 (7.68)	Untrained	IG: creative dance training, new theme every week (Magic House Task, Weather Task, Cowboy's and Indian's Task, Round the World Task)	5	IG: 3 CG: 3	IG: 45 CG: 45	(Paper-Pencil) Picture MRT (PMRT) with animals as stimuli (Neuburger et al., 2011): 1 set of 16 tasks; time-limit: 3 min. 2 items example and 2 items practice beforehand	Sig. main effect of group on MRT with larger pre-post-improvements in IG compared to CG; no. sig. effect of gender in IG and CG; no group × gender interaction
					CG: regular PE classes					MR performance correlated with intelligence, hand dexterity and M-ABC-2 score
									Movement Assessment Battery (M-ABC-2) (Petermann et al., 2009): 8 motor tasks: manual dexterity (3 tasks), ball skills (2 tasks), and static and dynamic balance (3 tasks)	No sig. main effect of group or gender on M-ABC-2; no group × gender interaction
									Number Connection Test (ZVT) (Oswald and Roth, 1987): 4 sheets; 60 s/sheet	No sig. main effect of group or gender on ZVT; no group × gender interaction
Pietsch et al. (2017)	46 (24/22)	23/23	n.s. (8.65)	Untrained	IG: Life Kinetik-motion program with cognitive, coordinative, and visual task complexes (clap–slap task, lateral and crossover motions, parallel balls, line jumping, finger game, course running, dancing balls, and circling eight) during PE class, some advancing in difficulty	5	IG: 2 CG: 2	IG: 20 CG: n/a	(Paper-Pencil) MRT with letters as stimuli (Quaiser-Pohl et al., 2014); [based on MRT by Vandenberg and Kuse (Vandenberg and Kuse, 1978)]: 16 tasks; time-limit: 2 min	Sig. main effect of time on MRT (pre-post-improvement); sig. time × group interaction with more sig. improvements in IG; sig. pre-post-improvement in IG; sig. between group difference at post-test
					CG: regular PE classes (tag and endurance games, ball games, and simple gymnastic routines)				ZVT (Oswald and Roth, 1987) (basline) Demographic Questionnaire in subsequent session	
Pietsch and Jansen (2018)	20 (10/10)	19/1	10–11 (10.60)	Trained (soccer)	IG: soccer specific tasks with non-dominant foot (designed to train mobilization, motor coordination, and dexterity for especially one foot)	10	IG: 1 CG: 1	IG: 30 CG:30	(Paper-Pencil) MRT with cube figures as stimuli (Quaiser-Pohl et al., 2014) [based on MRT by Vandenberg and Kuse (Vandenberg and Kuse, 1978)]: 16 tasks; time-limit: 2 min.	Sig. main effect of time on MRT (pre-post-improvement); sig. time × group interaction with sig. larger improvements in IG
					CG: same exercises with the dominant foot				ZVT (Oswald and Roth, 1987) (baseline)	Higher ZVT values in CG than IG at baseline
									Soccer Tests (based on testing manual for technomotor performance diagnostics of German soccer association): ball control: no. of passes in 30 s against bouncing wall from 5-m distance; dribbling: time dribbling through slalom course; shot precision: 6 shots, goal divided into zones with different strike values, 7-m distance Demographic Questionnaire	Sig. main effect of time on shot performance in IG (pre-post-improvement), not CG; sig. time × group interaction with larger improvements in IG compared to CG Sig. main effect of time on dribbling performance in IG (pre-post-improvement) and CG; no sig. time × group interaction for IG Sig. main effect of time on ball control in IG (pre-post-improvement), not CG; no sig. time × group interaction for IG and CG
**Visuo-Spatial working memory/spatial memory (Corsi block test; virtual reality morris water maze)**										
Notarnicola et al. (2012)	40 (20/20)	20/20	8–10 (9)	Untrained	IG: orienteering lessons with various activities (theory, practice and competitive aspects) including: learning to use maps, interpreting map contours, studying fieldwork techniques, cartography and designing maps, physical and environmental geography, measuring and locating shapes in space, estimating distance, determining direction and scale, handling data, creative writing, technical terms and symbols, making own orienteering equipment, building three-dimensional models from contour maps; orienteering exercises aimed at memory training (i.e., running while reading a story, then summarizing story at end of activity) CG: jogging training	26	IG: 3 (72 sessions) CG: 3 (72 sessions)	IG: n.s. CG: n.s.	Corsi Block Tapping Test (CBTT) (forward and backward): 4 trials Star-Butterfly Test (Kessels et al., 2000): time; no. of mistakes	Sig. between group differences on CBTT (forward and backward) at post-test in favor of IG; sig. pre-post-improvement on CBTT (backward) in IG Sig. between group differences on Star-Butterfly Test (time and mistakes) at post-test in favor of IG; sig. pre-post-improvement on Star-Butterfly Test in IG
Boccia et al. (2017)	34 (17/17)	19/15	4–6 (5.26)	Untrained	IG: navigational training program with paper-and-pencil and navigational activities (mental rotation, visuo-spatial and navigational memory, navigational planning, spatial orientation, left and right discrimination, spatial representation of body; each promoting landmark, route, and survey knowledge) CG: standard didactics (no additional training)	12	IG: n.s. (18 sessions) CG: n/a	IG: 90 CG: n/a	Walking Corsi Test (WalCT) with and without landmarks (LM), immediate and delayed response (Piccardi et al., 2008, 2014a,b): up to 18 trials (reaching route criterion) Landmark and survey knowledge tasks with LM-pictures from L-WalCT, route knowledge from WalCT and L-WalCT: error rates	Sig. main effect of time on topographical learning in WalCT with and without LM (pre-post-improvement); no sig. effects for topographical delayed recall in WalCT with and without LM No sig. effects on LM recognition; sig. main effect of time on path drawing in WalCT with and without LM (pre-post-improvement); sig. group × time interaction for LM location performance with sig. between group difference at post-test in favor of IG; sig. pre-post-improvement for LM location in IG
									Test for Reception of Grammar (TROG) (Bishop, 1982) (Italian version: (Chilosi and Cipriani, 1995)): 14 sentences; error rate Raven's Colored Progressive Matrices (Raven, 1986; Belacchi et al., 2008) (baseline)	Sig. main effect of time on TROG (pre-post-improvement); no sig. main effect of group; no sig. time × group interaction
Ben-Zeev et al. (2020)	40 (20/20)	40/0	12–13	Untrained	IG: high intensity functional training (HIFT) (dynamic warmup; multi-joint functional movements performed at high intensity, i.e., anaerobic exercises, and bodyweight/free-weight exercises; stretching cooldown) CG: regular PE-class (moderate-intensity cardiovascular exercises including jogging and sports games)	13	IG: 3 CG: 3	IG: 35 CG: 45	Virtual Reality Morris Water Maze (VMWM) (3 stages with four 35 s trials each): success rate; latency to reach target; path efficiency	Sig. improvement for success rate (stage 1 vs. stage 3) in IG, not CG at post-test; no between group differences at post-test sig. improvement for latency (stage 1 vs. stage 3) in IG, not CG at post-test; no between group differences at post-test sig. improvement for path efficiency (trial 1 vs. trial 4 at stage 3) in IG, not CG at post-test pre-post-improvement in movement plots (focusing around target) in IG, not CG
									Mnemonic Similarity Task:192 photographs with 64 repeated items; behavioral pattern separation performance; recognition score	Sig. pre-post-improvement for pattern separation in IG, not CG; no sig. between group differences at post-test no between group differences for recognition score
									Stroop Test (240 words in four colors): speed-accuracy tradeoff score calculated from reaction time and inhibitory control success rate;	Sig. pre-post-improvement for speed-accuracy tradeoff score in IG, not CG; no sig. between group differences at post-test
Latino et al. (2021)	60 (30/30)	30/30	14–15 (14.4)	untrained	IG: coordinative ability training designed to improve cognitive skills (slalom circuits, dexterity circuits, jump rope, throwing and catching, static and dynamic balance, jumps and direction changes, rhythm exercises, hand-eye and foot-eye coordination, motor response	12	IG: 2 CG: 2	IG: 60 (40 min. activity time) CG: 40	CBTT (Kessels et al., 2000): longest number of items reproduced at least twice Slalom Bask Test (Donati, 1994): time Throwing and Catching Test (Buonaccorsi, 2001): 10 valid throws and catches; time	Sig. time × group interaction for CBTT with sig. pre-post-improvements in IG, not CG No sig. time × group interaction for Slalom Bask Task Sig. time × group interaction for Throwing and Catching Test with sig. pre-post-improvements in IG, not CG
					exercises, motor differentiation exercises) CG: general psycho-physical wellness program (body-weight exercises, group exercises with small training gear, joint mobility, Calisthenics, Pilates)					
**Spatial orientation (Running-Orientation test)**										
Dirksen et al. (2015)	224 (113/111) after drop-out of classes: 107 (53/54) Analysis: coordina-tion: 91 (42/49) Writing and mathe-matics: 94 (47/47) Reading: 89 (47/42)		n.s. (12)	Untrained	IG: explicit movement-coordination exercises during PE class (complex tasks due to adaptation of sensory information or adding stressors) focused coordinative-cognitive, balance-, visuo-spatial- and differentiation-tasks CG: regular PE class (no additional training)	20	IG: 2 CG: n.a.	IG: 15 CG: n.a.	Spatial Orientation (run to numbered balls; 3 consecutive trials): time, no. of errors	Sig. main effect of time on spatial orientation; no sig. main effect of group; no sig. group × time interaction
									Balance (single leg stance on T-bar for 1 min.): time	Sig. main effect of time on balance; no sig. main effect of group; no sig. group × time interaction
									Eye-Hand-Coordination (single hand catching for 30 s): no. of successful catches	Sig. main effect of time on Eye-Hand-Coordination (pre-post-improvement); no sig. main effect of group; sig. group × time interaction in favor of IG with sig. pre-post-improvements in IG and CG
									Kinaesthetic Differentiation (throwing on target; different goal-zones in 4-m distance): no. of points	No sig. main effect of time and group on Kinaesthetic Differentiation; sig. group × time interaction in favor of IG with sig. pre-post-improvements in IG, not CG; sig. between group differences at post-test in favor of IG
									Reading [Salzburger Lesescreening 5–8 (Wimmer and Mayringer, 2002)]: read and judge correctness of sentences; time-limit: 3 min.	No sig. main effect of time or group; sig. group × time interaction on reading with sig. pre-post-improvement in CG and sig. pre-post-deterioration in IG; no sig. between group differences at pre- or post-test
									Writing (Hamburger Schreibprobe 5–9 (May, 2002)): error rate	Sig. main effect of time on writing (pre-post-improvement); no sig. main effect of group; sig. group × time interaction in favor of IG with sig. pre-post-improvement in IG; sig. between group difference at post-test in favor of IG
									Mathematics (newly developed test; Zinter, 2012, unpublished): score; time-limit: 30 min.	Sig. main effect of time on mathematics (pre-post-improvement); no sig. main effect of group; no sig. group × time interaction
Boraczyński et al. (2019)	75 (26/27/22)	75/0	10.1–11.8 (n.s.)	Trained (soccer) CG not involved in regular sports	IG1: on-field proprioceptive–coordinative (P-C) training (multi-mode P-C exercises with increasing intensity to substitute small-sided conditioning games in regular training); 30 min. before soccer practice (based on Polish Soccer Association for youth soccer-guidelines)	51	IG 1: 3 IG 2: 3 CG: n.a.	IG1: 90 IG2: 90 CG: n.a.	Tests for soccer-specific movement performance: -Movement rhythm (turning the ball backwards) -Motor adaptation (running with the ball around poles)	Sig. main effect of group on movement rhythm in favor of IG1 and IG2; no sig. main effect of time; no sig. group × time interaction Sig. main effect of group on motor adaptation in favor of IG1; no sig. main effect of time; no sig. group × time interaction
					IG2: regular training (based on Polish Soccer Association for youth soccer-guidelines)				-Spatial orientation (running to sequentially numbered balls)	Sig. main effect of group on spatial orientation in favor of IG1; sig. main effect of time with pre-post-improvement in IG1; no sig. group × time interaction
					CG: no intervention				-Balance (single-leg static balance)	Sig. main effect of group on balance in favor of IG1; sig. main effect of time with pre-post-improvement in IG1; sig. group × time interaction with higher and comparable pretest scores for IG1 and IG2 as opposed to CG and sig. higher peri- and post-test scores for IG1 compared to IG2
									-Kinesthetic differentiation of movement (landing the ball on a 2 × 2 m sector): time; accuracy -Heart rate during training (1 session/week; heart rate monitors)	Sig. main effect of group on kinesthetic differentiation in favor of IG1; sig. main effect of time with pre-post-improvement in IG1; no sig. group × time interaction

*CBTT, Corsi Block Tapping Test; CG, control group; f, female; HIFT, high intensity functional training; IG, intervention group; LM, landmark; m, male; M-ABC-2, Movement Assessment Battery; MRT, Mental Rotation Test; N, number of participants; n/a, not applicable; n.s., not stated; P-C, proprioceptive-coordinative; PE, physical education; PMRT, Picture Mental Rotation Test; RT, reaction time; TD, training duration (min/session); TF, training frequency (sessions/week); TP, training period (weeks); TROG, Test for Reception of Grammar; VMWM, Virtual Reality Morris Water Maze; WalCT, Walking Corsi Test; ZVT, Number Connection Test (Zahlenverbindungstest)*.

### Participants Characteristics

Summarizing participants from all included records, a total of 621 children and adolescents took part in the studies. All participants were healthy students with ages ranging from 4 to 15 years. While one study investigated 4–6-year-olds (Boccia et al., 2017), one study 12–13-year-olds (Ben-Zeev et al., 2020) and one study 14–15-year-olds (Latino et al., 2021), the mean age in the remaining studies ranged from 7.6 to 12.0 years. One record contained girls only (Jansen et al., 2011a), two boys only (Boraczyński et al., 2019; Ben-Zeev et al., 2020), one study had a male-female ratio of 19 to one (Pietsch and Jansen, 2018) and one did not give details on the amount of boys and girls participating but performed their intervention in regular school classes (Dirksen et al., 2015). The remaining studies had a more balanced gender distribution.

### Intervention Characteristics

Of all included studies, three implemented their training with participants engaged in regular sporting activities (i.e., soccer (Pietsch and Jansen, 2018; Boraczyński et al., 2019) or gymnastics (Jansen et al., 2011a)) while all other studies were conducted with regular school classes. Physical exercise programs that were eligible for inclusion comprised sport-specific activities (i.e., juggling, soccer and creative dance training) (Jansen et al., 2011a, 2013; Pietsch and Jansen, 2018), motor-coordinative exercises (i.e., Life Kinetik motion program, coordinative motor training or multi-mode proprioceptive-coordinative training) (Bluechel et al., 2013; Dirksen et al., 2015; Pietsch et al., 2017; Boraczyński et al., 2019; Latino et al., 2021), functional exercises at high intensity (Ben-Zeev et al., 2020), and training programs focusing on orientation and navigation (i.e., orienteering or navigation games) (Notarnicola et al., 2012; Boccia et al., 2017). Training programs were either conducted in a sports club (Jansen et al., 2011a; Boraczyński et al., 2019) or within a school setting (Notarnicola et al., 2012; Bluechel et al., 2013; Jansen et al., 2013; Dirksen et al., 2015; Boccia et al., 2017; Pietsch et al., 2017; Pietsch and Jansen, 2018; Ben-Zeev et al., 2020; Latino et al., 2021). They were delivered by gymnastic instructors (Jansen et al., 2011a), school teachers qualified as soccer coaches (Pietsch and Jansen, 2018), PE-teachers (Dirksen et al., 2015; Latino et al., 2021) or experimenters/trained instructors (Bluechel et al., 2013; Boccia et al., 2017; Ben-Zeev et al., 2020). Trainers were not stated explicitly in three trials (Notarnicola et al., 2012; Jansen et al., 2013; Boraczyński et al., 2019). Overall intervention duration and intensity varied widely across the included studies, ranging from 2 weeks to 12 months of training. Ten to 15 sessions amounting to a total of 200–675 min training were conducted in four trials (Bluechel et al., 2013; Jansen et al., 2013; Pietsch et al., 2017; Pietsch and Jansen, 2018). Five studies carried out 18 to 40 training sessions with a total of 600–1,620 min of training (Jansen et al., 2011a; Dirksen et al., 2015; Boccia et al., 2017; Ben-Zeev et al., 2020; Latino et al., 2021) and two studies trained for 72 (Notarnicola et al., 2012) and 117 sessions (Boraczyński et al., 2019), respectively, with only the latter one stating the total training time of 10,585 min.

### Outcome Measures

**Figure 2:**
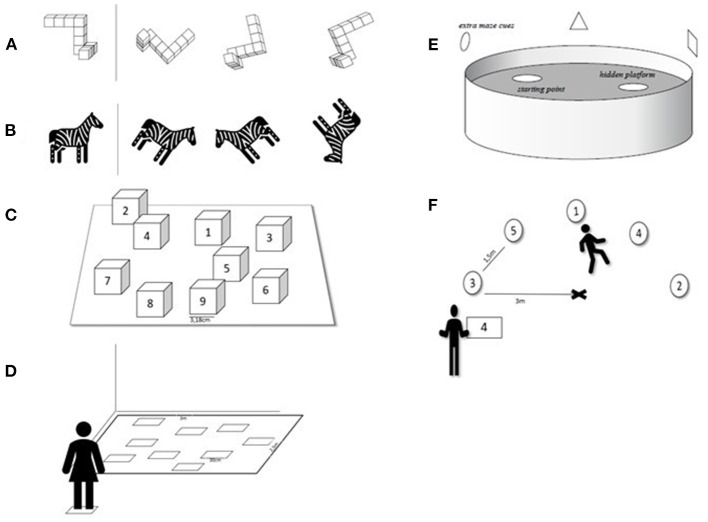
Illustrations of the main outcome measures. Mental rotation: **(A)** Mental Rotation Test with three-dimensional block figures, **(B)** Picture Mental Rotation Test; visuo-spatial working memory/spatial memory: **(C)** Corsi Block Tapping Test, **(D)** Walking Corsi Test, **(E)** Virtual Reality Morris Water Maze; spatial orientation: **(F)** Running-Orientation Test.

All records employed one main spatial outcome measure with five studies applying a Mental Rotation Test to evaluate MR performance (Jansen et al., 2011a, 2013; Bluechel et al., 2013; Pietsch et al., 2017; Pietsch and Jansen, 2018), three studies utilizing a type of the Corsi Block Test to assess visuo-spatial working memory (Notarnicola et al., 2012; Boccia et al., 2017; Latino et al., 2021), one study using a Virtual Reality Morris Water Maze to evaluate spatial memory (Ben-Zeev et al., 2020), and two studies using a Running-Orientation Test as a measure of spatial orientation (Dirksen et al., 2015; Boraczyński et al., 2019). Examples of the main spatial outcome measures are depicted in Figure 2.

### Effects on Mental Rotation

Three studies reported significant improvements in MR performance at post-test compared to pretest measurements with larger increases after sport-specific or motor-coordinative training (i.e., soccer specific training with the non-dominant leg, specific coordinative motor training or Life Kinetik training) compared to their respective control interventions (i.e., soccer specific training with the dominant leg, regular PE-class or regular class) (Bluechel et al., 2013; Pietsch et al., 2017; Pietsch and Jansen, 2018). While comparable at baseline, post-test scores differed significantly between intervention and control group in favor of motor coordination training (Bluechel et al., 2013; Pietsch et al., 2017). Differences between groups were not statistically significant in the study by Pietsch and Jansen (2018). Further, Jansen et al. (2013) found that the creative dance training group yielded better Mental Rotation Test results than regular PE-class group, similarly with larger performance increases from pre- to post-test after creative dance training compared to the control intervention. Moreover, MR appeared to correlate with intelligence as well as hand dexterity and overall scores on the Movement Assessment Battery for Children (M-ABC-2). In another study (Jansen et al., 2011a), these authors on the other hand, did not find any improvements in error rate neither after juggling nor following light strength training. When analyzing only the correct responses, they did, however, report significantly faster reaction times in the Mental Rotation Test at 90° and 180° rotation after juggling training compared to the strength training group. In relation to other rotational angles, the highest reaction time improvements were observed at 90° for the juggling group.

### Effects on Visuo-Spatial Working Memory/Spatial Memory

The three studies evaluating visuo-spatial working memory made use of different versions of the Corsi Block Test, that is the Corsi Block Tapping Test (CBTT) and the Walking Corsi Test (WalCT) in two conditions (i.e., with and without landmarks). Notarnicola et al. (2012) and Latino et al. (2021), however, reported on increasingly long tapping sequences, while Boccia et al. (2017) made use of fixed supra-span sequences and related to a learning criterion aiming at three consecutively correct reproductions of a given sequence. With scores in the forward and backward CBTT comparable at pretest, Notarnicola et al. (2012) reported significant between group differences at post-test in favor of the orienteering group compared to the jogging group. Moreover, the orienteering group significantly improved their performance from pre- to post-test, whereas these changes were not observed in the jogging group. Similarly, Latino et al. (2021) observed a significant improvement from pre- to post-test in the coordination-training group only compared to a general psycho-physical wellness program. In the third study, topographical learning in the WalCT with and without landmarks improved significantly between pre- and post-test (Boccia et al., 2017). With ceiling effects at pretest, no significant pre-post-differences could be established for topographical delayed recall of the test-sequence. The study investigating spatial memory evaluated the success rate to find a hidden platform, latency to reach the platform and path efficiency in a Virtual Reality Morris Water Maze (VMWM) (Ben-Zeev et al., 2020). While performance at pretest was similar for both groups, the group performing high intensity functional training (HIFT) was able to significantly improve their achievements from pre- to post-test on all three parameters. The group following regular PE-class showed no significant amelioration. Only the HIFT-group was able to concentrate their movement significantly closer to the hidden platform at post-test compared to pretest measurements as can be seen by a movement plot. However, no significant differences were observed between both groups at post-test for success rate and latency to reach the target area.

### Effects on Spatial Orientation

In the studies evaluating spatial orientation by means of running tests, Dirksen et al. (2015) observed significant advances in speed at post-test compared to baseline measurements, however development between groups did not differ significantly. In contrast, Boraczyński et al. (2019) reported overall better results in the proprioceptive-coordinative training group with only this group significantly improving their orientation test speed from pre- to post-test.

### Moderator Variables

With regards to moderator variables, one study identified a significant positive correlation between age and training effects in the juggling as well as the strength training group with participants' age ranging from 6 to 14 years (Jansen et al., 2011a). Two further trials evaluated sex-effects on their outcomes (Bluechel et al., 2013; Jansen et al., 2013). Bluechel et al. (2013) found a significant main effect of sex in 8-10-year-old children with higher Mental Rotation Test scores for boys. This was not supported by the findings of Jansen et al. (2013) in a comparable age group (7 to 9 year old children). None of the included studies investigated effect of physical training status on their results.

## Discussion

This systematic scoping review provides an overview over the current body of evidence regarding the impact of physical exercise interventions on spatial abilities of healthy children and adolescents. Overall, there appears to be potential for the improvement of spatial abilities in a young and healthy population by means of physical exercise interventions. Evidence, however, remains limited as it becomes obvious how widely the included studies' approaches differ in terms of study design, population characteristics, choice of intervention, training parameters and outcome measures.

### Key Measures, Key Elements of Physical Exercise, and General Findings

In accordance with the literature, MR was the outcome measured most frequently in the included studies. With the Mental Rotation Test as an easy to administer and well-described tool, in depth investigations of this spatial ability has been conducted ongoing since the 1970s (Shepard and Metzler, 1971; Vandenberg and Kuse, 1978). With multiple modifications in place (i.e., choice of stimuli, rotation angles, number of target stimuli, time limitations) (Quaiser-Pohl, 2003; Voyer and Hou, 2006) the Mental Rotation Test has been utilized throughout various age- and population groups (Rüsseler et al., 2006; Jansen and Heil, 2010). Four of the five studies in this review administrating a Mental Rotation Test report improvements in MR performance in favor of the respective intervention group (Bluechel et al., 2013; Jansen et al., 2013; Pietsch et al., 2017; Pietsch and Jansen, 2018). Even though test stimuli differed (i.e. 3-D block figures, animal pictures, letters), all of these studies applied a similar test methodology presenting one target item and four sample items. Out of those sample items, participants had to identify the two items that were identical but rotated in relation to the target item. Twelve to 16 tasks had to be solved within a time limit of 2 to 3 min depending on the study. In contrast, the one study that did not observe MR improvements followed a different approach (Jansen et al., 2011a). Participants had to compare one target and one sample stimulus (i.e., 3-D block figures) and decide whether the items were identical or different. That is rotated or rotated and mirror reversed. Four-hundred and thirty-two tasks were performed in ~50 min. These authors did, however, report faster reaction times in favor of the intervention group when analyzing only correct responses. It should be noted that participants in this trial were specifically instructed to answer “as quickly and accurately as possible.” Therefore, the methodologically different approach by Jansen et al. (2011a) combined with the explicit focus on speed during test conduction might have shaped their findings for the benefit of speed rather than accuracy. While it is known that the choice of stimuli impacts on MR performance between different age groups (i.e., children, young adults, elderly adults) (Iachini et al., 2019), it appears that within the studied age group (mean age: 7.7–10.6 years) the choice of stimuli was not decisive for MR performance. It is noteworthy that improvements were observed in those studies administering cognitively more demanding and complex tasks (i.e., finding two correct items out of four) in contrast to slightly less demanding “same or different” -decisions (Titze et al., 2010) even though a time-limitation was set. While participants in the study by Jansen et al. (2011a) trained for a slightly longer time period (i.e., 13 weeks), training modalities in the remaining MR-studies were similar (i.e., up to 10 weeks), which implicates that longer training periods might not necessarily result in superior MR performance. Three studies reporting enhanced MR conducted motor-coordinative training programs or contained vast amounts of motor-coordination exercises (Bluechel et al., 2013; Pietsch et al., 2017; Pietsch and Jansen, 2018). These findings are in line with studies by Jansen and Heil (2010) who found a relation between MR performance and motor tasks involving coordinative aspects of 5 and 6 year old children and Pietsch and Jansen (2012), who report a correlation between MR and motor-coordinative abilities in young adults. As juggling might also be considered an intervention with high motor-coordinative requirements (Zelic et al., 2012), results from the study by Jansen et al. (2011a) could be viewed from this point of view as well. Since these authors, did not find notable improvements in MR performance, one might consider that the study population were gymnasts. Studies in adult populations show that gymnasts or athletes performing sports that involve significant amounts of mental rotation (i.e., wrestling) perform better on MR tasks than non-athletes or athletes in sports not involving MR (i.e., running) (Moreau et al., 2012; Jansen and Lehmann, 2013). Therefore, MR performance at baseline might have already been higher than in some of the other studies recruiting students from schools potentially limiting the range for enhancement. This could further explain why creative dance training resulted in superior MR achievements than regular PE-class (Jansen et al., 2013). To gain more insight into the factors that influence MR performance in children and adolescents it would be of value to develop contrasting study designs that compare e.g., the same physical exercise intervention for different periods of time, compare participants involved in regular sporting activities with those who do not exercise regularly or compare two promising training interventions e.g., coordinative vs. sport-specific exercises within one study in the future.

The Corsi Block Test, a second well-established outcome measure that aims to evaluate visuo-spatial working memory, was applied by three studies in this review (Corsi, 1973). Even though the CBTT used by Notarnicola et al. (2012) and Latino et al. (2021) as well as the WalCT conducted by Boccia et al. (2017) have been used in other studies (Farrell Pagulayan et al., 2006; Piccardi et al., 2014b), results must be interpreted with care. In line with Farrell Pagulayan et al. (2006) and Berch et al. (1998), the modifications of various task aspects and discrepancies in the implementation of the task diminish the comparability and reproducibility of this test. Both studies employing the CBTT report significantly longer tapping sequences following their intervention condition (i.e., orienteering lessons or coordination training) compared to the respective control condition (Notarnicola et al., 2012; Latino et al., 2021). Even though methodology in both studies differed slightly (i.e., initial sequence of two vs. three blocks; forward and backward sequences vs. forward only) overall implementation of the test was alike. In the WalCT administered by Boccia et al. (2017) children hat to reproduce a fixed supra-span sequence relating to their age for three consecutive times. Here, significant improvements in both groups were detected. It should, however, be noted that participants in the latter study were considerably younger (mean age: 5.26 years) than those in the former two studies (mean age: 9 and 14.4 years). These findings are backed by Piccardi et al. (2014b) who compared the performance on the CBTT with the WalCT in 4–11-year-olds. The authors report slow but steady improvements with increasing age on both tests with superior performance on spatial span tasks (i.e., CBTT) compared to navigational span tasks (i.e., WalCT) from the age of six onwards. In younger children, test performance in both conditions was comparable, indicating that important spatial developmental progresses are made around this age. The effect of training modalities and the type of training on spatial outcomes remains inconclusive. The combination of motoric and cognitive-navigational exercises resulted in superior CBTT performance in the study by Notarnicola et al. (2012), not, though, in WalCT advances in the study by Boccia et al. (2017). Yet, training frequency differed considerably between studies with 72 sessions for the former and 18 sessions for the latter. Again, a coordinative training approach as implemented by Latino et al. (2021) provides promising results. Future research is needed to investigate in more depth into e.g., the effect of one physical exercise intervention on the CBTT in comparison to the WalCT, the effect of a physical exercise program on these working memory tasks comparing different age groups, or the comparison of different physical exercise approaches (e.g., coordination vs. a combination of motor and cognitive exercises) within a specific age group.

The Morris Water Maze has extensively been used and validated as a tool to assess spatial learning and spatial memory in rodents (Morris, 1981; Daugherty et al., 2015). Analogous virtual reality versions of this measure have been developed and successfully applied in human research throughout all age groups (Newhouse et al., 2007; Daugherty et al., 2015). However, experimental procedures and designs are not standardized across human studies (Commins et al., 2020). Although significant improvements were reported for all parameters tested in the VRMWM after HIFT compared to regular PE-class (Ben-Zeev et al., 2020), adequate comparable studies in humans are still lacking to date. Even though, Herting and Nagel (2012) demonstrate enhanced spatial learning behavior on a Morris Water Maze in male adolescents with higher aerobic fitness levels, future research is needed to validate these first promising results.

The Running-Orientation Test conducted in the studies by Dirksen et al. (2015) and Boraczyński et al. (2019) receives little attention in the international scientific literature and appears to be utilized predominantly as a subtest in coordination-assessments in the German-speaking region (Hirtz, 1985; Glasauer, 2003; Golle et al., 2019), which might limit diagnostic conclusiveness of this outcome measure. Study population, type of intervention, training modalities, test execution and findings differ considerably between both studies impeding further appraisal of confounding factors on this test.

On the whole, motor-coordination exercises appear to be a key parameter in the development of spatial abilities of children and adolescents. The five studies in this review focusing their physical exercise interventions predominantly on motor-coordination programs report overall improvements from pretest to post-test in their respective spatial outcome measure with the intervention groups showing larger improvements than the control groups in four of the five studies (Bluechel et al., 2013; Pietsch et al., 2017; Boraczyński et al., 2019; Latino et al., 2021). Comparable to the intervention by Boraczyński et al. (2019), a significant part of laterality specific soccer training conducted in the study by Pietsch and Jansen (2018) was made up of coordination exercises, yielding similar results to the motor-coordination interventions mentioned previously. In light of additional research supporting this finding (Jansen and Heil, 2010; Pietsch and Jansen, 2012), large, controlled and reproducible studies are needed to investigate the promising effect of motor-coordination training on different spatial abilities, on different age-groups, on different sexes as well as on participants with different training status.

In summary, the included studies indicate that physical exercise, particularly with coordinative elements, has a facilitating effect on the development and improvement of spatial abilities of children and adolescents.

### Role of Moderator Variables

While motor activity might have played an important role in enhancing spatial abilities, improvements in some studies occurred irrespective of training group (Dirksen et al., 2015; Boccia et al., 2017). This could be related to the general human growth and maturation process, as it is well-known that different aspects of spatial abilities develop with increasing age. Mental rotation performance for example that is considerably above the level of chance, appears to be evident from around the age of five with significant skill development between the ages of three and five (Funk et al., 2005; Frick et al., 2013b). Enhanced MR performance with increasing age can subsequently be observed in 5 to 7 year old children (Fernandez-Baizan et al., 2020), with comparable findings holding true for other spatial tasks like navigational place learning or topographic memory (Leplow et al., 2003; Piccardi et al., 2014b). The Morris Water Maze as well as the Radial-Arm-Maze, another relevant measure to evaluate the development of these spatial abilities, have been developed for rodents and have pre-dominantly been studied in adult populations to date. Several studies applying variations of these tests in younger age groups, however, establish that spatial learning and spatial memory are not fully developed until at least around the age of seven in humans with crucial progressions taking place between the ages of 5 and 7 (Overman et al., 1996; Leplow et al., 2003; Mandolesi et al., 2009; Fernandez-Baizan et al., 2021). Moreover, progressive, age-related improvements in these skills have been demonstrated from childhood throughout adolescence to early adulthood by various studies utilizing adaptations of the Morris Water Maze (Piper et al., 2010; Sneider et al., 2015; Fernandez-Baizan et al., 2021). A study by Sneider et al. (2015) revealed that even though all participants improved their performance on a virtual Morris Water Maze, young adults (mean age: 21.6 years) demonstrated faster learning and superior retention of the target area as opposed to adolescents (mean age: 13.6 years). It has been well-established that spatial memory as measured by the Morris Water Maze deteriorates again with increasing age throughout adulthood (Moffat and Resnick, 2002; Schoenfeld et al., 2014). Farrell Pagulayan et al. (2006) further discovered that spatial span capacity on the Corsi Block Test increased gradually with age, when comparing a group of children (mean age: 7 years) with adolescents (mean age: 14 years) and young adults (mean age: 21 years). At the same time, they found that results of adolescents and young adults did not differ significantly, leading to the hypothesis that adult-like spatial capacity is reached throughout adolescence. These findings are supported by Frick et al. (2009), who found 11 year old children's and adults' reaction time on a Mental Rotation Test to be unrelated to a matching or mismatching manual rotation task as opposed to 5 and 8 year old children. Their performance was better when manual rotation and MR directions matched. In this present review, only one study took a closer look at age as an independent influential factor on spatial abilities (Jansen et al., 2011a). In line with previous findings, training effects of both juggling and strength training correlated with age in 6 to 14 year old girls, while only age, but not motor performance, significantly affected MR reaction time in the intervention group (Jansen et al., 2011a). Unfortunately, no further differentiations of the age-related findings were provided in the respective study and participants' ages covered a large span.

Associated to age and maturation, sex-effects, hormonal status, and changes in hormonal balance during puberty ought to be discussed as potential confounders of spatial abilities. Two studies in this review reflected on sex-effects in relation to their outcome measures (Bluechel et al., 2013; Jansen et al., 2013) with contradictory results. While a significant effect of sex in favor of boys was observed in the Mental Rotation Test in children aged 8 to 10 years by Bluechel et al. (2013), these findings were not supported by Jansen et al. (2013), who conducted a Mental Rotation Test in a similar age group and did not find significant differences between girls and boys. Literature on the effects of sex on spatial abilities is manifold but inconsistent. The assumption that sex differences in spatial abilities are not present throughout childhood but start to emerge from puberty onwards and increase with age are endorsed by various studies (Voyer et al., 1995; Piccardi et al., 2008, 2014b). Nonetheless, differences between boys and girls in favor of male participants have been found in MR at primary school age by Rodan et al. (2019) when controlling for intelligence as well as by Newhouse et al. (2007) on a virtual Morris Water Maze task. In a comparable age group, Tzuriel and Egozi (2010) found that girls were able to nearly disperse the gender-gap that was present on a MR task at baseline after participating in a cognitive intervention focusing on spatial abilities. At the same time, some studies involving pubertal or post-pubertal populations did not find sex-related discrepancies (Leplow et al., 2003; Farrell Pagulayan et al., 2006; Rodan et al., 2016). Several recent meta-analyses have further addressed sex differences and spatial abilities throughout different age groups. Lauer et al. (2019) evaluated sex differences in MR performance of 3-17-year-olds. Analysis revealed that a performance gap in favor of males slowly starts to occur in the first years of school and continues to grow till late adolescence independent of procedural moderators. When exploring on the navigational skills of males and females, Nazareth et al. (2019) revealed that males significantly outperformed females on a variety of navigational paradigms. Having said that, effect sizes were significantly smaller in studies with participants below the age of 13 compared to other age groups, indicating that sex differences increase from puberty onwards. Gender-specific performance on visuo-spatial working memory tasks on the other hand first begins to emerge around puberty. While Voyer et al. (2017) report significantly better outcomes for males on these tasks in general, age appears to be a significant moderator. Nevertheless, the underlying mechanisms for these gender disparities remain unclear. Explanations for sex divergence on spatial abilities are widespread. Jansen-Osmann and Heil (2007) attempt to break them down to two mayor concepts, namely psych-social and biological-neuronal factors, which involve amongst others socialization and upbringing, gender stereotypes, hormonal status, or neurological maturation. Instead of true differences between males and females, heterogeneity in spatial outcome measures, test administration and methodological inconsistencies or sensitivity of measures within a certain age-group have been discussed as causative parameters as well, challenging the explanatory power of sex-discrepancies on spatial tests (Voyer et al., 1995; Farrell Pagulayan et al., 2006). Moreover, it has been suggested that the occurrences of sex-differences might not be established with one spatial outcome measure alone but that spatial abilities are represented by a range of skills that relate to participants' age in different ways (Fernandez-Mendez et al., 2018).

### Strengths and Limitations of the Present Scoping Review

A systematic scoping review of the literature was conducted to map available evidence and identify knowledge gaps regarding the effect of physical exercise interventions on spatial abilities in healthy children and adolescents. Eleven studies met our predefined eligibility criteria and were included in this scoping review. The limited availability of studies investigating on this effect by means of a controlled study design with pre- and post-measurements indicates that this field has not been researched exhaustively to date in this specific population. Study designs, population characteristics, intervention approaches, training parameters, and outcome measures varied significantly across the included studies. In line with the scope of this review, a descriptive analysis of the results was performed accordingly. Despite methodological differences of the included studies, overall a positive impact of physical exercise interventions on spatial abilities of studied population can be derived from the findings.

Only studies published in English or German were included in this systematic scoping review. Since English is the dominating language in scientific literature, it was expected that the vast majority of research findings to date are available in this language. However, due to this language bias, additional evidence from well-done studies published in other languages might have been missed. Including studies published in other languages could have added further value to this review. Even though the literature search conducted for this scoping review was performed in a structured and careful manner, it cannot be ruled out that eligible publications were not detected based on the search strategy applied.

At the same time, this scoping review followed a thorough methodological regimen as specified by the PRISMA-guidelines for scoping reviews (Tricco et al., 2018). The large-scale search of the literature that was conducted, allows for the assumption that all relevant literature on this topic was detected and scrutinized for inclusion. Moreover, the detailed appraisal of moderating variables on spatial abilities in addition to a summary of the main findings adds significant value to this scoping review.

With respect to study results as well as strengths and limitations, several research gaps were identified. Overall, it can be said that large scale, methodologically sound studies are needed to pursue promising approaches identified in this research. Future research should e.g. compare the effect of a motor-coordinative exercise intervention on spatial abilities with a sport-specific training requiring high degrees of MR, a training regimen requiring no MR or no training. Moreover, addressing the impact of a specific physical exercise intervention on a variety of spatial abilities like MR, visuo-spatial working memory or spatial memory and spatial orientation or navigation abilities within one study will help to understand what kind of exercise interventions are needed to impacts on each of these skills.

To acquire a deeper insight into the current understanding of spatial ability development throughout childhood and adolescence this future research should be conducted under consideration of various moderating variables. As already attempted by some authors who compared different age groups in their studies, it is of importance to continue and extend the existing research base on age-related developmental factors that influence spatial performance (Farrell Pagulayan et al., 2006; Frick et al., 2009). It is a great challenge for researchers to differentiate systematically and consistently, which type of spatial abilities start to emerge from which age onwards and which extrinsic (i.e., education, social and economic background) and intrinsic (i.e., intelligence, hormonal status or general motor and/or cognitive development) variables might impact on this development. To date, knowledge on the impact of sex on spatial abilities in children and adolescents is limited and inconsistent. It is therefore necessary to continue researching in more detail which underlying processes and factors might cause differences in spatial activities and/or spatial outcomes between males and females and more specifically between boys and girls. In light of previous efforts (Voyer et al., 1995, 2017; Levine et al., 2016), it is inevitable to survey the variables age and sex in conjunction with one another as influential factors like prenatal hormonal exposure, changes in hormonal status in puberty as well as experience (i.e., playing), exposure to gender-stereotypes and resulting self-conception are discussed to impact on spatial performance while growing up (Quaiser-Pohl et al., 2014; Levine et al., 2016; Reilly et al., 2017). In line with the aim of this research, physical training status is a third potentially moderating factor to consider in future research. Only one study in this review compared participants involved in regular sporting activity with those that are not (Boraczyński et al., 2019). Unfortunately, the untrained participants served as a no-training control group. Therefore, no conclusions could be drawn regarding the impact of a physical training intervention on spatial abilities in these participants with different physical training levels. At the same time, studies in young adults indicate a superior Mental Rotation Test performance of athletes who performed their respective sport (i.e., orienteering, gymnastics, running, rugby, handball, soccer, basketball, badminton, judo) at least twice per week compared to non-athletes (Ozel et al., 2002; Schmidt et al., 2016). Moreover, it was detected, that not only training status but also the type of sport performed might have an impact on MR abilities. Sports with higher cognitive demands or three-dimensional axial activities (i.e., orienteering, gymnastics, wrestling) resulted in higher Mental Rotation Test scores as opposed to those sporting activities requiring less cognitive involvement (i.e., running) (Moreau et al., 2012; Schmidt et al., 2016). With larger improvements in MR reaction time after juggling compared to light strength training in girls, these findings are supported by Jansen et al. (2011a). As the underlying processes are not completely understood yet, they should be evaluated further in upcoming research.

This review only included studies with healthy young populations. In light of equal opportunities (i.e., the global rise of obesity as well as the efforts of many countries to implement inclusive education), it might be of interest to gain deeper insights into the relation of motor abilities and spatial abilities of children with overweight and obesity as well as with developmental disorders. Emerging gaps in spatial abilities should be detected as early as possible in order to develop appropriate countermeasures and counteract this development. According to Jansen et al. (2011b), overweight and obese children showed lower motor performance and MR ability on MR-tasks with larger rotation angles compared to normal weight children. These findings were extended by Deconinck et al. (2019), who reported constraints in MR of obese children with additional motor impairments but not in obese children without motor impairment compared to normal weight children. Yet, the relationship between motor and cognitive development is not as clear cut in children with developmental disorders. Opposite to expectations, Farran et al. (2019) did not detect impairments in large-scale spatial cognition measured with a virtual environment maze navigation task in children with attention deficit hyperactivity disorder compared to typically developing children, despite the former group presenting with impairments in motor tasks. Individuals with Williams Syndrome on the other hand showed lower performance in both domains. This could indicate pathways for the formation of spatial abilities next to motor development.

In order to facilitate spatial ability development in the best possible way, it would further be worthwhile to gain deeper insights into ideal training parameters of physical exercise interventions in this context. According to the World Health Organization (2010), children and adolescents between the age of five and 17 should on average perform at least 60 min moderate to vigorous physical activity on a daily basis with vigorous activities at least three times per week. While overall recommendations on motor-coordination training parameters are limited, somewhat more specific recommendations on strength (Dahab and McCambridge, 2009; Granacher et al., 2009; Lesinski et al., 2016), balance (Gebel et al., 2018) or endurance (Armstrong and Barker, 2011) training during childhood and youth exist for the respective motor performances. In addition, some limited evidence exists on the beneficial effect of performing organized sport with regard to improvement in motor skills including coordination and fitness in school-aged children (i.e., 6–12 years) (Vandorpe et al., 2012; Vallence et al., 2019). However, guidelines of physical training parameters in relation to spatial abilities in children and adolescents do not exist. Trainings in the included studies covered a broad range of frequencies, intensities, and durations with only three studies giving a very limited justification for their choice (Notarnicola et al., 2012; Dirksen et al., 2015; Boraczyński et al., 2019). It can be presumed that decisions were at least partially fitted to the preconditions of the respective study environments. Four of the nine studies conducted in a school environment were realized within the regular PE-classes of the students. Three of these studies (Jansen et al., 2013; Dirksen et al., 2015; Pietsch et al., 2017) were conducted in Germany and in line with the school curriculum recommending two to three PE-classes per week (Landesstelle für den Schulsport, 2020), one study was conducted in Israel (Ben-Zeev et al., 2020). For the studies conducted in a sports-club one can assume, that training frequencies were in line with the regular training regimens. Even though all included studies showed some spatial ability improvements, regarding the large variety in training regimens and training parameters and a lack of literature on this topic, the most favorable training parameters for spatial ability training by physical exercise interventions cannot be specified. Yet, given the fact that children and adolescents spend the majority of their time within the school-context, it would be useful to consider how such measures could be implement on a regular basis within the school environment (i.e., PE or extracurricular activities). This would open up an ideal opportunity for educators to efficiently encourage children's and adolescents' motor and cognitive skill development at the same time.

## Conclusions

Spatial abilities are considered to have a substantial impact on various aspects of leading an independent and successful life. Even though the relation between cognitive and motor development in children and adolescents appears to be well-established, little is known about the effect physical exercise has on complex cognitive functions like spatial abilities. This systematic scoping review reveals promising trend of various physical exercise interventions on the formation and enhancement of spatial abilities in young and healthy populations. In particular, motor coordinative training programs appear to facilitate spatial development. In light of the high potential for adaptation, learning, and plasticity of the young body and brain it is essential for future research to improve comprehension of this topic and to identify how these developments can be supported in the best possible way. The current body of evidence indicates the great opportunity that physical fitness, motor skills and spatial abilities can be addressed with the same targeted training regimen. Amongst others it should therefore be established in more depth at what age which spatial abilities form and how they can be promoted by physical exercise, whether there is a specific timeslot to impact on potential sex differences or which type of exercise and which training parameters have the highest impact on spatial abilities in this population.

## Author Contributions

This systematic scoping review was carried out collaboratively by both authors. The systematic literature search was conducted independently by CM and TM. Both authors screened the titles and abstracts of all results for eligibility according to the inclusion and exclusion criteria and assessed full-text versions of all potentially relevant studies for inclusion. CM wrote the first draft of the manuscript. Both authors have been involved in the drafting and contributed significantly to the revision of this manuscript. All authors approved the final version.

## Conflict of Interest

The authors declare that the research was conducted in the absence of any commercial or financial relationships that could be construed as a potential conflict of interest.

## Supplementary Material

Supplementary Material 1 Example of the final search string for the Web of Science database.

Supplementary Material 2 Prisma checklist.

## Abbreviations

CBTT: Corsi Block Tapping Test
HIFT: high intensity functional training
M-ABC-2: Movement Assessment Battery for Children
MR: mental rotation
PE: physical education
VMWM: Virtual Reality Morris Water Maze
WalCT: Walking Corsi Test
ZVT: Number Connection Test (Zahlenverbindungstest).
