# Supplementary material for: Effects of Physical Exercise Interventions on Spatial Orientation in Children and Adolescents: A Systematic Scoping Review
Source: Front Sports Act Living. 2021 Jun 17;3:664640. doi: 10.3389/fspor.2021.664640 (PMC8247469; doi:10.3389/fspor.2021.664640)
Supplement: Supplementary Material 1 — Example of the final search string for the Web of Science database. [file Data_Sheet_1.docx]

**Supplement 1:** Example of the final search string for the Web of Science database

ALL FIELDS: ("spatial navigat*") OR ALL FIELDS: ("spatial orientat*") OR ALL FIELDS: ("spatial behavio*r") OR ALL FIELDS: (wayfinding) OR ALL FIELDS: ("route learning") OR ALL FIELDS: ("mental* rotati*") OR ALL FIELDS: ("cognitive map*") OR ALL FIELDS: (allocentric*) OR ALL FIELDS: (egocentric*)

ALL FIELDS: (intervention*) OR ALL FIELDS: (exercise*) OR ALL FIELDS: (training*) OR ALL FIELDS: ("motor* training*") OR ALL FIELDS: ("physical* training*")

ALL FIELDS: (child*) OR ALL FIELDS: (adolescent*) OR ALL FIELDS: (youth) OR ALL FIELDS: ("primary school") OR ALL FIELDS: ("secondary school")

ALL FIELDS: (Spatial navigat* abilit*) OR ALL FIELDS: (spatial orientat* abilit*) OR ALL FIELDS: ("spatial learning") OR ALL FIELDS: (wayfinding) OR ALL FIELDS: ("route learning") OR ALL FIELDS: ("mental* rotati*") OR ALL FIELDS: ("spatial skill*") OR ALL FIELDS: ("motor* activit*") OR ALL FIELDS: ("physical fitness") OR ALL FIELDS: ("motor skill*") OR ALL FIELDS: (coordinat*) OR ALL FIELDS: (academic performance) OR ALL FIELDS: (school achievement*) OR ALL FIELDS: (grade*)

#4 AND #3 AND #2 AND #1
